# Persistent Liver Dysfunction in Pediatric Patients After Total Cavopulmonary Connection Surgery

**DOI:** 10.3389/fcvm.2022.820791

**Published:** 2022-04-26

**Authors:** Qipeng Luo, Yuan Jia, Zhanhao Su, Hongbai Wang, Yinan Li, Xie Wu, Qiao Liu, Xiaoguang Liu, Su Yuan, Fuxia Yan

**Affiliations:** ^1^Pain Medicine Center, Peking University Third Hospital, Peking University Health Science Center, Beijing, China; ^2^Department of Anesthesiology, Fuwai Hospital, National Center of Cardiovascular Diseases, Chinese Academy of Medical Sciences and Peking Union Medical College, Beijing, China; ^3^Center for Pediatric Cardiac Surgery, Fuwai Hospital, National Center of Cardiovascular Diseases, Chinese Academy of Medical Sciences and Peking Union Medical College, Beijing, China

**Keywords:** anesthesia, congenital heart disease, pediatric cardiac surgery, total cavopulmonary connection surgery, liver dysfunction, central venous pressure, risk assessment

## Abstract

**Background:**

Studies have reported early liver dysfunction (LD) after cardiac surgery is associated with short and long-term mortality. In this study, we aimed to investigate risk factors for persistent LD after total cavopulmonary connection (TCPC) surgery.

**Methods:**

This is a retrospective case-control study. We defined persistent LD as LDs occurring between postoperative day 1 (POD1) and POD7 and sustaining at least on POD7, while transient LD as LDs occurring between POD1 and POD7 and recovering at least on POD7. Multivariable logistic regression analysis was applied and central venous pressure (CVP) was considered continuously or in quantiles.

**Results:**

Postoperative LD occurred in 111 (27.1%) patients. Transient and persistent LD occurred in 65 (15.9%) and 46 (11.2%) patients, respectively. Aortic cross-clamping (ACC) (odds ratio [OR] 2.55, 95% CI 1.26–5.14) and postoperative CVP (OR 1.34, 95% CI 1.18–1.51) were risk factors for persistent LD, also identified for postoperative any LD and transient LD. Adding postoperative CVP to the model only including ACC significantly improved persistent LD prediction (△AUC 0.15, *p* = 0.002). Compared with CVP ≤ 14 mmHg, adjusted ORs and 95% CI of persistent LD for CVP of 14–16 and >16 mmHg were 3.11 (1.24, 7.81) and 10.55 (3.72, 29.93), respectively. Patients with persistent LD might have a longer length of mechanical ventilation (mean difference, 13.5 h) and postoperative hospital stay (mean difference, 7 days), and higher postoperative costs (mean difference, 6.7 thousand dollars) compared to those with transient LD.

**Conclusions:**

Intra-operative application of ACC and postoperative elevated CVP were independent risk factors for persistent LD in pediatric patients following TCPC surgery. Compared to patients with transient LD, patients with persistent LD might have a longer length of mechanical ventilation and postoperative hospital stay, and higher postoperative costs. We should pay more attention to patients with high postoperative CVP to prevent their persistent LD occurrence.

## Introduction

The liver is usually a “forgotten” organ in the perioperative management of patients undergoing congenital the heart surgery when compared to the kidney. Complex cardiac lesions such as single ventricles have certain anatomic and hemodynamic features that predispose these patients to hepatic dysfunction (1). Hepatic function abnormalities can even be seen before and early after total cavopulmonary connection (TCPC) surgery (2), due to the unique circulatory characteristics including elevated central venous pressure (CVP) and impaired cardiac output (3).

As ScienceDaily’s science news reported in 2017, all patients will suffer from some liver diseases of different degrees in severity, which is just a matter of time, and the severity degrees of these liver diseases become poorer over time since Fontan or TCPC procedure (4). The risk of developing liver cirrhosis, one of the most severe liver diseases, is as high as 43% in 30-year follow-up after Fontan operation (5). Therefore, Fontan-associated liver disease has become an important issue both early and late after Fontan surgery, and positive actions including paying attention to postoperative liver dysfunction and timely identifying its risk factors should be taken to reduce progressive deterioration of liver function after TCPC surgery.

Previous studies reported that the incidence of the liver dysfunction (LD) after open-heart surgery was as high as 25.6% in neonate patients (6), even up to 53.3% in adult patients (7). LD after adult cardiac surgery was associated with prolonged length of mechanical ventilation, hospital stay, and even in-hospital and long-term mortality (8). Some risk factors for hepatic dysfunction after adult cardiovascular surgery have also been identified, such as low cardiac ejection fraction, aortic cross-clamping (ACC) time, cardiopulmonary bypass (CPB) time, etc. (9, 10). Furthermore, a recent study of a large sample size (*n* = 11, 198) reported that postoperative initial CVP of >11 mmHg was associated with a higher risk for postoperative liver dysfunction and in-hospital mortality in adult patients after cardiac surgery (11). But the relationship between high CVP and LD after TCPC surgery remains unclear and there is also no report about the risk factors for LD in patients after TCPC surgery. To address these current knowledge gaps, this study was designed to identify risk factors for LD and the relationship between postoperative CVP and LD in 409 children undergoing TCPC surgery.

## Materials and Methods

### Study Population

All data were from the Fuwai TCPC cohort (12). Pediatric patients under 12 years old who underwent TCPC surgery were consecutively included. Patients were excluded if they died or had extracorporeal membrane oxygenation or had missing values of postoperative alanine aminotransferase (ALT) or aspartate aminotransferase (AST). This is a retrospective observational study. Informed consent was waived because this study was a retrospective study.

### Definition of the Liver Dysfunction

We operationally defined LD as postoperative ALT or AST ≥ 2 times above the normal upper limit according to the definition of LD in a previous study published in the Journal of the American College Cardiology in 2019 (6). The diagnosis of LD between postoperative day 1 (POD1) and POD7 was based on the maximal ALT or AST levels that were collected between POD1 and POD7. We defined transient LD as patients who developed LD between POD1 and POD7 and did not have LD between POD7 and POD14 or POD7 and discharge. Patients, who developed LD between POD1 and POD7 and still had LD between POD7 and POD14 or POD7 and discharge, were defined as having persistent LD.

### Variable Definition and Collection

Preoperative peripheral oxygen saturation was recorded when inhaling air at the time of admission to the hospital. The echo data (ventricle ejection fraction and ventricle end-diastolic diameter) were measured in the main ventricle. We defined senior surgeons as surgeons who performed an average of TCPC procedures over 10 cases per year. The mean arterial blood pressure and the corresponding CVP were the mean of the first six measurements (one measurement per 30 min) after admission to the intensive care unit (ICU).

Fluid balance (ml/kg) was calculated: fluid balance = [(amount of crystalloids + colloids + red cell + plasma + platelets + priming volume) - (blood loss + urine output + ultrafiltrate + residual blood in CPB machine)]/weight. *Z*-scores indexed to body surface area were applied when calculating the main ventricular end-diastolic diameter z-score. The estimated glomerular filtration rate was computed according to the previous study (13). Vasoactive-inotropic score (VIS) was referred to in the previous formula (14).

Other perioperative variables were also collected, which included gender, age at operation, weight, history of prior cardiovascular surgery, and anatomical diagnosis, Nakata index from cardiovascular CT or catheterization, pulmonary artery pressure from catheter data (pulmonary vascular resistance not reported in all catheter reports, but pulmonary artery pressure did) or from internal jugular venous pressure if patients with prior Glenn surgery, echo data, preoperative blood biomarkers (blood routine test, liver and renal function test, and isoenzyme of creatine kinase-MB), usage of dexamethasone, platelet transfusion, CPB time, and POD0 maximal lactate.

Indications for fenestration included unexpected conditions that occurred during the operation, heterotaxy syndrome, postoperative CVP > 15 mmHg, CPB > 150 min, concomitant atrioventricular valve surgery, pulmonary vascular resistance > 4U/m^2^ or average pulmonary artery pressure > 15 mmHg, McGoon ratio < 1.8 or pulmonary artery index < 250 mm^2^/m^2^, asymmetric development of pulmonary arteries, moderate or above cardiac dysfunction, and non-sinus rhythm.

### In-Hospital Clinical Outcomes

The in-hospital outcomes included duration of ICU stay and postoperative hospital stay, length of mechanical ventilation, renal replacement treatment, and postoperative hospitalization costs (conversion: 1 US dollar ≈ 6.5 RMB). Renal replacement treatment included peritoneal dialysis and CRRT.

### Statistical Analysis

The statistical analysis was approved by Institutional Review Board. The R package “missForest” with a random forest algorithm was used to impute the missing data. Differences in the distribution of data before and after imputation were evaluated. Student’s *t*-test or Mann-Whitney *U* tests were used to analyze quantitative data, and *X*^2^ or Fisher exact tests were used to analyze categorical data, as appropriate, these statistic methods were used to test the significance of the difference between transient and no LD or persistent and no LD or persistent and transient LD.

Postoperative CVP was included in analysis continuously or in quantiles of 0.75 and 0.9. Collinear relationships between potential risk factors were performed before multivariate analysis. Univariate logistic regression and multivariate logistic regression analyses using the backward elimination stepwise method were applied to identify risk factors for any LD, transient LD, and persistent LD. Multivariate logistic regression analysis was started with variables with *p* ≤ 0.2 in univariate logistic regression analysis. The adjusted odds ratio (OR) and 95% CI were obtained through multivariate logistic regression analysis with the entrance of all univariables (*p* ≤ 0.2 in univariate logistic regression analysis).

The calibration curve and Hosmer–Lemeshow goodness-of-fit test were used to assess the fitness or calibration of models, and the receiver operating characteristic curve and the areas under receiver operating curves (AUCs) were used to evaluate the discrimination ability of the logistic models. We used the DeLong’s method to compare the AUCs of two model predictions (15). Two subgroups (age ≤ 4 years old and prior Glenn surgery) were operationally selected to examine whether the logistic models can be applied to a subgroup populations of different characteristics, *via* calculating Hosmer–Lemeshow goodness-of-fit test and AUCs. *p* ≤ 0.05 was considered statistically significant. All analyses were performed in R software (version 3.6.4).

## Results

### Characteristics of the Study Population

**FIGURE 1:**
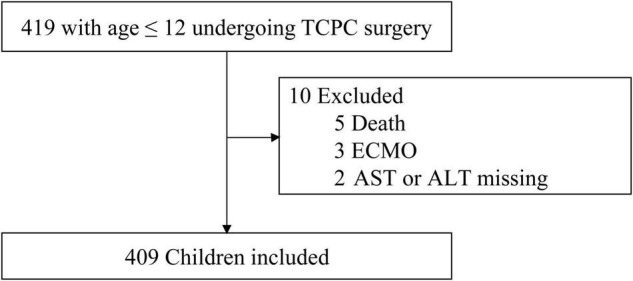
The flowchart of inclusion and exclusion criteria.

From July 2010 to June 2019, a total of 419 children patients (≤12 years old) underwent TCPC surgery. In total, five children who died during hospitalization were excluded because of missing serum creatinine, and also complicated and uncontrolled confounders in these patients, one of the five deaths had a normal ALT or AST between POD1 and POD7 but had LD on POD10. Three children with ECMO after TCPC were excluded due to dilution of ALT and AST within the circuit. Two patients were excluded because of missing ALT and AST. After the exclusion criteria, 409 cases were included in the final analysis. The flow chart is shown in Figure 1. The rate of missing data in this study population ranged from 0.2 to 3.9%. Complete data were seen in 372 (90.5%) cases. Among the identified risk factors included in the final logistic models, no one had missing data (Supplementary Table 1). The differences in distributions among data before and after imputation were not significant.

The median and interquartile range of the age and weight were 5.3 (4, 7.4) years old and 18 (15, 21) kg, respectively. The top three diagnoses in these patients were an unbalanced atrioventricular septal defect, tricuspid atresia, and unbalanced double outlet right ventricle. Twenty-six patients (6.4%) had a diagnosis with heterotaxy syndrome and 17.9% of patients with unbalanced atrioventricular septal defects were with isomerism. Most of the patients (97.1%; *n* = 397) underwent extracardiac TCPC and the other 12 patients underwent internal tunnel TCPC. The percentages of right and left ventricle morphology were 41.8 and 39.1%, respectively. All patients underwent CPB and 29.1% (*n* = 119) patients were performed with ACC. A percentage of 68% patients had prior Glenn surgery (*N* = 278) and 4.6% patients had prior B–T shunt (*N* = 19). The baseline characteristics and perioperative data are displayed in Table 1. No patients had preoperative ALT or AST ≥ 2 times above normal the upper limit.

**TABLE 1:** Patient characteristics and perioperative information according to the liver function development.

Variables	No-LD (*n* = 298)	Transient LD (*n* = 65)	Persistent LD (*n* = 46)	Overall (*n* = 409)
Gender (male)	191 (64.1%)	34 (52.3%)	32 (69.6%)	257 (62.8%)
Age (years)	5.3 (4.0, 7.1)	5.0 (3.7, 7.0)	5.6 (4.0, 9.0)	5.3 (4.0, 7.4)
Weight (kg)	18.0 (15.0, 21.0)	17.5 (15.0, 21.0)	17.5 (15.5, 23.8)	18.0 (15.0, 21.0)
Preoperative SpO2 (≤80%)	101 (33.9%)	23 (35.4%)	15 (32.6%)	139 (34.0%)
Priori Glenn surgery n (%)	198 (66.4%)	50 (76.9%)	30 (65.2%)	278 (68.0%)
Priori B-T shunt surgery n (%)	11 (3.7%)	6 (9.2%)	2 (4.3%)	19 (4.6%)
Heterotaxy syndrome diagnosis n (%)	16 (5.4%)	5 (7.7%)	5 (10.9%)	26 (6.4%)
Ventricle morphology n (%)				
Intermediated	51 (17.1%)	18 (27.7%)	9 (19.6%)	78 (19.1%)
Left	121 (40.6%)	19 (29.2%)	20 (43.5%)	160 (39.1%)
Right	126 (42.3%)	28 (43.1%)	17 (37.0%)	171 (41.8%)
Anatomical diagnosis n (%)				
Unbalanced AVSD	74 (24.8%)	27 (41.5%)	11 (23.9%)	112 (27.4%)
Tricuspid atresia	73 (24.5%)	6 (9.2%)	10 (21.7%)	89 (21.8%)
Unbalanced DORV	59 (19.8%)	11 (16.9%)	9 (19.6%)	79 (19.3%)
Double inlet ventricle	25 (8.4%)	1 (1.5%)	7 (15.2%)	33 (8.1%)
Pulmonary atresia	24 (8.1%)	12 (18.5%)	4 (8.7%)	40 (9.8%)
Others	43 (14.4%)	8 (12.3%)	5 (10.9%)	56 (13.7%)
Nakata index	196 (140, 256)	172 (132, 223)	159 (116, 195)	188 (135, 245)
Pulmonary artery pressure (mmHg)	11.4 ± 3.4	11.4 ± 3.3	11.7 ± 3.3	11.5 ± 3.4
Main ventricle EF (<55%)	39 (13.1%)	6 (9.2%)	2 (4.3%)	47 (11.5%)
Main ventricle EDDz	−0.4 (−4.8, 2.8)	−1.7 (−4.3, 2.2)	−1.4 (−5.6, 2.0)	−1.1 (−4.8, 2.7)
Preoperative biomarkers				
Hemoglobin (g/L)	17.6 ± 2.7	17.7 ± 2.5	17.6 ± 2.4	17.6 ± 2.6
Albumin (g/L)	44.2 ± 3.3	44.0 ± 3.9	44.2 ± 3.9	44.2 ± 3.5
Platelet count (×10^9^/L)	253 ± 85	275 ± 91	267 ± 83	258 ± 86
ALT (U/L)	15 (11, 20)	14 (11, 18)	16 (13, 19)	15 (11, 20)
AST (U/L)	28.2 ± 7.6	29.7 ± 7.3	28.2 ± 7.5	28.4 ± 7.5
Total bilirubin (mg/dl)	0.77 (0.58, 1.05)	0.78 (0.53, 1.35)	0.75 (0.61, 1.04)	0.77 (0.58, 1.08)
eGFR (mL/min/1.73 m^2^)	103 (92.0, 115)	98.6 (89.7, 115)	101 (93.1, 117)	101 (91.5, 117)
CK-MB (IU/L)	24 (20, 33)	26 (20, 32)	26 (22, 31)	25 (20, 33)
Anesthesia and operation				
Senior surgeons n (%)	192 (64.4%)	43 (66.2%)	29 (63.0%)	264 (64.5%)
VISm	10.9 ± 6.5	16.1 ± 8.8*	13.6 ± 8.8*	12.0 ± 7.5
Use of dexamethasone n (%)	260 (87.2%)	51 (78.5%)	43 (93.5%)^†^	354 (86.6%)
Platete transfusion *n* (%)	176 (59.1%)	51 (78.5%)*	27 (58.7%)^†^	254 (62.1%)
CPB time (min)	108 ± 43.4	146 ± 57.2*	131 ± 67.5*	117 ± 51.1
Aortic cross-clamp n (%)	65 (21.8%)	37 (56.9%)*	17 (37.0%)*^†^	119 (29.1%)
Fenestration n (%)	119 (39.9%)	30 (46.2%)	22 (47.8%)	171 (41.8%)
Fluid balance (mL/kg)	8.0 (−8.7, 24.0)	8.9 (−11.4, 21.0)	19.8 (−2.8, 35.4)*	8.9 (−8.5, 25.1)
POD0 maximal lactate (mmol/L)	2.08 ± 1.28	2.84 ± 1.72*	2.87 ± 2.21*	2.29 ± 1.52
POD0 CVP (mmHg)	12.1 ± 3.1	13.1 ± 3.0*	13.4 ± 3.3*	12.4 ± 3.1
POD0 MAP (mmHg)	60.4 ± 9.4	59.7 ± 11.5	59.3 ± 9.8	60.2 ± 9.8

LD, liver dysfunction; SpO2, peripheral oxygen saturation; ASVD, atrioventricular septal defect; DORV, double outlet right ventricle; EF, ejection fraction; EDDz, end-diastolic diameter z-score; ALT, alanine aminotransferase; AST, aspartate aminotransferase; eGFR, estimated glomerular filtration rate; CK-MB, isoenzyme of creatine kinase-MB; VISm, intraoperative maximum vasoactive inotropic score; CPB, cardiopulmonary bypass; POD0, postoperative day zero; CVP, central venous pressure; MAP, mean arterial blood pressure. Quantitative variables: means ± SD or median (interquartile range); categorical variables: frequency (percentage). *p ≤ 0.05 compared with no-LD group; ^†^p ≤ 0.05 compared with transient LD group.

### Risk Factors for the Liver Dysfunction

After multivariate analysis with backward elimination stepwise regression method and starting with variables of *p* ≤ 0.2 in univariate logistic regression analysis, primary diagnosis (tricuspid atresia as ref.; unbalanced atrioventricular septal defect OR 2.74[1.01, 7.5]; pulmonary atresia OR 4.57[1.4, 14.92]; unbalanced double outlet right ventricle OR 1.59[0.52, 4.91]; double inlet ventricle OR 0.34[0.03, 3.36]; pulmonary atresia OR 4.57[1.4, 14.92]; other types OR 1.51[0.44, 5.18]; LR-test *p* = 0.024), intra-operative maximal VIS (OR 1.05[1.01, 1.1]), CPB time (OR 1.01[1.002, 1.02]), ACC (OR 3.01[1.53, 5.93]), and postoperative CVP (OR 1.16[1.04, 1.29]) were independent predictors for transient LD, which derived from multivariate logistic regression analysis with the comparison between transient LD group and no-LD group, while application of ACC (OR 2.55[1.26, 5.14]) and postoperative CVP (OR 1.34[1.18, 1.51]) were identified as independent risk factors for persistent LD (Table 2), which derived from multivariate logistic regression analysis with the comparison between persistent LD group and no-LD group. Intra-operative maximal VIS (OR 1.05[1.02, 1.08]), CPB time (OR 1.01[1.007, 1.01]), ACC (OR 2.52[1.44, 4.43]), and postoperative CVP (OR 1.24[1.13, 1.35]) were independent risk factors for postoperative any type of LD.

**TABLE 2:** Univariate and multivariate logistic analysis for persistent liver dysfunction.

Variables	Univariate analysis		Multivariate analysis*	
		
	OR (95% CI)	*P*	OR (95% CI)	*P*
Weight (kg)	1.04(1,1.08)	0.065		
Heterotaxy syndrome (yes vs. no)	2.15(0.75,6.18)	0.181		
Nakata index	0.997(0.994,1.01)	0.143		
Main ventricle EF (<55%)	0.3(0.07,1.3)	0.058		
Pre-OP ALT (U/L)	1.03(1,1.06)	0.072		
Intra-OP maximal VIS	1.05(1.01,1.09)	0.018		
Intra-OP dexamethasone (yes vs. no)	2.09(0.62,7.09)	0.194		
Intra-OP fluid balance (ml/kg)	1.01(1,1.03)	0.038		
CPB time (min)	1.0083(1.0028,1.014)	0.004		
Aortic cross-clamp (yes vs. no)	2.1(1.09,4.06)	0.031	2.55 (1.26, 5.14)	0.01
POD0 maximal lactate (mmol/L)	1.32(1.1,1.6)	0.002		
POD0 CVP (mmHg)	1.31(1.16,1.48)	0.001	1.34 (1.18, 1.51)	<0.001

OR, odds ratios; CI, confidence interval; OP, operative; ALT, alanine aminotransferase; VIS, intraoperative maximum vasoactive inotropic score; CPB, cardiopulmonary bypass; POD0, postoperative day zero; CVP, central venous pressure. *Multivariate analysis with backward elimination stepwise regression method and starting with variables of p ≤ 0.2 in univariate logistic regression analysis.

Postoperative CVP was divided into three segments using 0.75 and 0.9 quantiles of CVP as the two cutoffs, namely, 14 and 16 mmHg. The same 5 risk factors were identified for transient LD and the same 2 risk factors for persistent LD. After adjustment of gender, priori Glenn surgery, ventricle morphology, anatomical diagnosis, AST, total bilirubin, intra-operative maximal VIS, use of dexamethasome, transfusion of platelet, CPB time, ACC, and POD0 maximal lactate (*P* ≤ 0.2 in univariate logistic regression analysis), the adjusted ORs and 95% CIs of transient LD for CVPs of 14–16 and >16 mmHg were respectively 1.23 (1.001, 2.9) and 2.78 (1.16, 9.23) using CVPs at ≤ 14 mmHg as reference. After adjustment of weight, diagnosis of heterotaxy syndrome, Nakata index, main ventricle ejection fraction, ALT, intra-operative maximal VIS, use of dexamethasone, intra-operative fluid balance, CPB time, ACC, POD0 maximal lactate (*P* ≤ 0.2 in univariate logistic regression analysis), the adjusted ORs and 95% CIs of persistent LD for CVPs of 14–16 and >16 mmHg were, respectively, 3.11 (1.24, 7.81) and 10.55 (3.72, 29.93) using CVPs at ≤14 mmHg as reference. After adjustment of weight, prior B–T shunt surgery, diagnosis of heterotaxy syndrome, ventricle morphology, anatomical diagnosis, Nakata index, main ventricle ejection fraction, platelet counts, total bilirubin, intra-operative maximal VIS, transfusion of platelet, CPB time, ACC and POD0 maximal lactate, the adjusted ORs and 95% CIs of any LD for CVPs of 14–16 and >16 mmHg were, respectively, 2.05 (1.06, 3.98) and 7.43 (3.07, 17.99) using CVPs at ≤14 mmHg as reference. The adjusted ORs are presented in Table 3.

**TABLE 3:** Univariate and multivariate logistic analysis of the association between postoperative CVP and postoperative any type of LD, transient LD and persistent LD.

Events	CVP (mmHg) in quantiles/Continuously	Number and rate of events n (%)	Crude OR (95% CI)	*P*	Adjusted OR* (95% CI)	*P*
Any type of LD (*n* = 111)	≤14	69 (21.3%)	Reference	-	Reference	-
	14–16	22 (39.3%)	2.31 (1.27, 4.19)	0.006	2.05 (1.06, 3.98)	0.033
	>16	20 (69.0%)	5.83 (2.63, 12.91)	<0.001	7.43 (3.07, 17.99)	<0.001
	Continuously	-	1.22 (1.12, 1.33)	<0.001	1.21 (1.11, 1.33)	<0.001
Transient LD (*n* = 65)	≤14	45 (13.9%)	Reference	-	Reference	-
	14–16	12 (21.4%)	1.90 (1.001, 3.93)	0.048	1.23 (1.001, 2.90)	0.038
	>16	8 (27.6%)	2.94 (1.04, 8.33)	0.043	2.78 (1.16, 9.23)	0.009
	Continuously	-	1.17 (1.06, 1.30)	0.002	1.15 (1.02, 1.31)	0.029
Persistent LD (*n* = 46)	≤14	24 (7.41%)	Reference	-	Reference	-
	14–16	10 (17.9%)	3.1 (1.37, 7.04)	0.007	3.11 (1.24, 7.81)	0.016
	>16	12 (41.4%)	11.5 (4.59, 28.83)	<0.001	10.55 (3.72, 29.93)	<0.001
	Continuously	-	1.31 (1.16, 1.48)	<0.001	1.26 (1.10, 1.45)	<0.001

OR, odds ratios; CI, confidence interval; CVP, central venous pressure; LD, liver dysfunction. *With adjustment for variables of p ≤ 0.2 in univariate logistic regression analysis.

### Performance of the Persistent Liver Dysfunction Models

**FIGURE 2:**
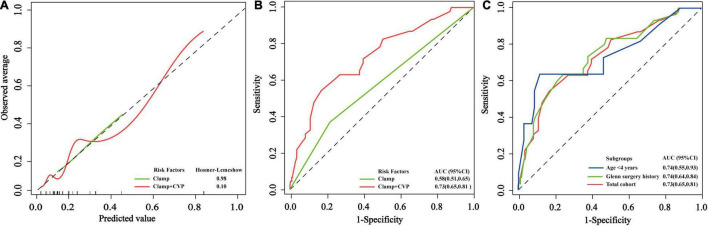
The performance of the logistic regression models for persistent LD. **(A)** Calibration curves and corresponding *p*-values from Hosmer–Lemeshow goodness-of-fit test in the total cohort. **(B)** The receiver operating characteristic curves and their areas under receiver operating curves in the total cohort. **(C)** The receiver operating characteristic curves were used for evaluating the performance of the persistent LD logistic regression model in the two subgroups with age ≤4 years old and prior Glenn’s surgery.

The model only including preoperative variables identified no factors for persistent LD. When adding the intra-operative variables, the model identified ACC as the risk factor for persistent LD and its AUC was 0.58 (95% CI, 0.51–0.65). When further addition of postoperative CVP to the model only including ACC, the discrimination power was notably improved (△AUC, 0.15; DeLong *p* = 0.002). The AUC was 0.73 (95% CI, 0.65–0.81) in the model including these two factors. The calibration and ROC curves are displayed in Figures 2A,B. The AUCs for persistent LD in the subgroup with age ≤4 years old and prior Glenn subgroup were 0.74 (0.55, 0.93) and 0.74 (0.64, 0.84), respectively (Figure 2C). The corresponding Hosmer–Lemeshow test *p* > 0.05.

### Postoperative In-Hospital Clinical Outcomes

One hundred and eleven patients (27.1%, 111/409) developed LD between POD1 and POD7 of TCPC surgery and 58.6% (65/111) of LDs were transient LD (15.9% of the total cohort). Among the 409 patients, 46 patients (11.2%) had persistent LD. The median and interquartile range of postoperative hospital stay of the total cohort was 19 (13, 34) days. The median and interquartile range of postoperative hospital stay in patients with transient and persistent LD were 21 (15, 33) and 28 (17, 40) days, respectively, with a significant increase in longer hospital stay of an average of 7 days in persistent LD group.

Among 278 patients with prior Glenn procedures, 86 patients (30.9%, 86/278) had ACC, which accounted for 21% of the total cohort. Compared to patients with no ACC, patients with ACC had a higher rate of transient LD (31.1 vs. 9.7%) and persistent LD (14.3 vs. 10%).

We divided CVPs into three segments using two cutoffs (14, 16 mmHg), namely, 0.75, 0.9 quantiles of CVPs. Persistent LD rates in patients with CVPs at ≤14, 14–16, >16 mmHg were 7.4, 17.9, 41.4%, respectively, while transient LD rates among those three segments of the CVP group were 13.9, 21.4, 27.6%, respectively (Table 3).

A percentage of 41.8% of patients (*n* = 171) had creation of fenestration. There were no differences between the fenestration group and non-fenestration group for postoperative CVP, postoperative LD, length of mechanical ventilation, and use of renal replacement treatment (all *P* > 0.05). Compared with patients in non-fenestration group, the creation of fenestration might be associated with shorter postoperative hospital stay (median length 21 vs. 18 days, *p* = 0.029).

Compared with patients in the no LD group, the development of transient and persistent LD after TCPC surgery might be associated with a longer length of mechanical ventilation and postoperative hospital stay, a higher rate of renal replacement treatment, and higher postoperative hospitalization costs. Compared with transient LD, the development of persistent LD might be associated with a longer length of mechanical ventilation (30.5 vs. 17.0 h), postoperative hospital stay (28 vs. 21 days), and higher postoperative costs (28.3 vs. 21.6 thousand US dollars). In-hospital outcomes among no-LD, transient, and persistent LD are presented in Table 4.

**TABLE 4:** Postoperative short-term outcomes of the study patients.

Variables	No-LD (*n* = 298)	Transient LD (*n* = 65)	Persistent LD (*n* = 46)
ICU stay (days)	2 (1, 4)	4 (2, 8)	6 (2, 11)
Length of mechanical ventilation (hours)	10.0 (6.0, 19.0)	17.0 (9.0, 52.0)*	30.5 (19.0, 70.8)*^†^
Postoperative hospital stay (days)	17 (12, 32)	21 (15, 33)*	28 (17, 40)*^†^
Renal replacement treatment n (%)	12 (4.0%)	15 (23.1%)*	13 (28.3%)*
Postoperative costs (thousands US dollars)	16.8 ± 9.7	21.6 ± 11.1*	28.3 ± 14.2*^†^

LD, liver dysfunction; Quantitative variables: median (interquartile range); Categorical variables: frequency (percentage). *p-value ≤ 0.05 compared with no-LD group; ^†^p-value ≤ 0.05 compared with transient LD group. 1 US dollars ≈ 6.5 RMB.

## Discussion

This study demonstrated that the development of LD was common (27.1%, 111/409) and about half of them (46/111) worsen to persistent LD (11.2% of the total cohort, 46/409). Development of persistent LD was linked to poor in-hospital outcomes, especially to the length of mechanical ventilation, postoperative hospital stay, and postoperative hospitalization costs. Intra-operative application of ACC and postoperative CVP were identified as independent risk factors for any LD, transient LD, and persistent LD. Our findings are helpful to develop an early risk stratification for persistent LD in these vulnerable patients following TCPC surgery and improve their outcomes.

The incidence of postoperative LD varies under different criteria of LD and ranges from 19 to 53.3% (6, 7, 16–19). This study defined the LD on the basis of AST or ALT, which might underestimate the incidence of LD because we did not consider bilirubin in the definition of LD. Some studies defined LD as ALT or AST higher than the normal upper limit (7). The study in the Journal of the American College Cardiology defined LD as ALT or AST 2 times higher than the normal range, which was the criteria used in this study, and in which the incidence of LD was slightly lower than that in our study (25.6% vs. 27.1%) (6), this might be explained by more palliative surgery (all TCPC surgery) in our study, which was associated with a higher risk of hepatic injury when compared with other cardiac surgery (16). The incidence of LD was lower than that in a previous study (27.1 vs. 53.3%), in which LD was defined as ALT or AST higher than the normal upper limit (7). Furthermore, the definition of LD after cardiac surgery still needs further investigation, and statistically driven cutoffs of LD and a combination of liver ultrasonography, clinical scoring system, and other specific biomarkers might be a future direction in this area.

Previous studies have shown persistent organ injuries had more and more severe clinical outcomes, even long-term mortality when compared with transient organ injuries (17, 18). In our study, patients with persistent LD had longer lengths of mechanical ventilation and postoperative hospital stay, and higher postoperative costs when compared to those with transient LD, which suggested that persistent LD might have a more substantial influence on the clinical outcomes and we should pay more attention to the persistent LD.

Congestion is one of the fundamental factors for the development of liver injury and a relatively high CVP is essential to patients after TCPC surgery, which seems to be a paradox. A previous studies suggested CVP < 14 mmHg as management criteria in a Fontan circulation (19). In our study, postoperative CVP was an independent risk factor for both transient and persistent LD, and a postoperative CVP at 14–16 mmHg was associated with 3.1-fold higher risks of persistent LD compared with a CVP ≤ 14 mmHg, and risks of persistent LD for CVP at >16 mmHg increased to 10.6-fold. A previous study also indicated that better short-term outcomes were achieved in the sildenafil group with a relatively low mean pulmonary artery pressure after the Fontan operation (20). However, the optimal CVP cutoffs considering both high-CVP-related complications and Fontan circulation requirements, still need further study.

Aortic cross-clamping is often used in cardiac surgery and provides a good vision of the surgical field. In this study, patients with concomitant atrioventricular surgeries, pulmonary arterioplasty, and other circumstances such as torturous vessel, complex surgeries, severe adhesion, and so on, underwent the TCPC using ACC. Previous studies have identified ACC time is a risk factor for hepatic dysfunction in patients undergoing acute type A aortic dissection surgery (21), which was consistent with our results. In this study, the application of aortic cross-clamping was an independent risk factor for both transient and persistent LD, which might be attributed to increased inflammation response and impaired cardiac function in patients undergoing aortic cross-clamping. However, the concrete mechanism requires further investigation.

In this study, we obtained 12 univariables with *p* ≤ 0.2 for persistent LD *via* univariate logistic regression analysis. We performed multicollinearity analysis, and we also used multivariate logistic regression analysis with backward elimination stepwise regression method to avoid severe multicollinearity among these covariates. Eventually, the correlation coefficients of ACC and CPB time were 0.56 with *p* < 0.001, and the correlation coefficients of ACC and POD0 maximal lactate were 0.36 with *p* < 0.001. Other pair-wise correlation coefficients of these 12 univariables for persistent LD were <0.2 with *p* > 0.5. Intra-operative ACC and postoperative CVP were independent risk factors for persistent LD in the model adjusting eight variables after excluding CPB and POD0 maximal lactate. Furthermore, with all these 12 univariables entering logistic regression, only postoperative CVP was an independent risk factor, but when using multivariate logistic regression analysis with backward elimination stepwise regression method and starting with all these 12 univariables, intra-operative ACC and postoperative CVP were identified as two risk factors for persistent LD. Therefore, we used multiple regression methods (entering and stepwise regression methods) and subtypes of LDs (any type of LD, transient LD, and persistent LD) to increase the robustness of our results. However, the relationship among CPB time, POD0 maximal lactate, and persistent LD still needs to be studied.

Among 409 patients, 131 patients (32%) had no prior Glenn surgery before TCPC surgery and most of them were the cases in their early years (before 2014) and underwent single-stage TCPC without prior Glenn. The older operation age when coming to the hospital and the relatively poorer economic status in our country may drive patients or doctors to choose the single-staged TCPC (22). In our study, prior Glenn surgery was not a protective or risk factor for LD, and patients with prior Glenn history had severe adhesion after the first cardiac surgery and surgeons needed some time to release the adhesion under CPB in these patients. Despite these differences in the populations in our study, the models for persistent LD can be applied to the subgroups of age ≤4 years old and prior Glenn surgery and their AUCs were more than 0.7.

### Limitations

Our study had several limitations. First, the results from this study might be subject to updates of clinical practice, therefore no causal conclusion can be made from this retrospective study, the real relationship between perioperative LD and poor outcomes like liver fibrosis etc., still needs further study. Second, not all potential parameters were included, such as the velocity of hepatic artery flow, transient elastography, and cardiac magnetic resonance-derived metrics, because these variables were not routinely assessed when hospitalization. Third, our study was limited by failing to demonstrate that the recovery of liver function after POD14 or discharge because not all patients tested their liver function after POD14 or discharge, especially in patients with transient LD. Last, this study indicated that postoperative CVP was a robust risk factor for LD, but the exact cutoff of optimal postoperative CVP still needs further study.

## Conclusion

In Chinese pediatric patients after TCPC surgery, LD after TCPC surgery was a common complication. The incidence of persistent LD was as high as 11.2%. Intra-operative application of ACC and postoperative CVP were identified as independent risk factors for any LD, transient LD, and persistent LD. Postoperative LD was linked to poor short-term clinical outcomes, and patients with persistent LD had a longer lengths of mechanical ventilation and postoperative hospital stay, and higher postoperative costs when compared to those with transient LD. We should pay more attention to patients with higher postoperative CVP (especially > 14 mmHg) to reduce their persistent LD occurrence.

## Data Availability Statement

The original contributions presented in the study are included in the article/Supplementary Material, further inquiries can be directed to the corresponding author.

## Ethics Statement

The studies involving human participants were reviewed and approved by Ethics Committee of the Chinese Academy of Medical Sciences Fuwai Hospital. Written informed consent for participation was not provided by the participants’ legal guardians/next of kin because: this study was a retrospective study.

## Author Contributions

YJ, FY, XL, and SY conceived and designed the study. QPL, ZS, HW, YL, XW, and QL arranged, analyzed, and interpreted the data. QPL, ZS, YJ, SY, and FY drafted the manuscript or revised it critically. All authors contributed extensively to the work presented in this manuscript and read and approved the version to be submitted.

## Conflict of Interest

The authors declare that the research was conducted in the absence of any commercial or financial relationships that could be construed as a potential conflict of interest.

## Publisher’s Note

All claims expressed in this article are solely those of the authors and do not necessarily represent those of their affiliated organizations, or those of the publisher, the editors and the reviewers. Any product that may be evaluated in this article, or claim that may be made by its manufacturer, is not guaranteed or endorsed by the publisher.
