# Supplementary material for: Persistent Liver Dysfunction in Pediatric Patients After Total Cavopulmonary Connection Surgery
Source: Front Cardiovasc Med. 2022 Apr 26;9:820791. doi: 10.3389/fcvm.2022.820791 (PMC9087337; doi:10.3389/fcvm.2022.820791)
Supplement: Supplementary file 1 [file Table_1.DOCX]

**Supplemental Table 1. Missing measurements**

| **Variables** | **Total cohort (N=409)** | |  |
| --- | --- | --- | --- |
|  | **No.** | **Percentage (%)** | |
| **Preoperative pulmonary arterial pressure** | 16 | 3.9 |  |
| **Main ventricular ejection fraction** | 1 | 0.2 |  |
| **Main ventricular end-diastolic diameter z-score** | 8 | 2 |  |
| **Preoperative alanine aminotransferase** | 1 | 0.2 |  |
| **Preoperative aspartate aminotransferase** | 1 | 0.2 |  |
| **Preoperative total bilirubin** | 11 | 2.7 |  |
| **Postoperative day zero maximal lactic acid** | 8 | 2 |  |

Total missing value rate: 9.5%.
